# Risk factors for digital stress in German public administrations

**DOI:** 10.1186/s12889-021-12247-w

**Published:** 2021-12-03

**Authors:** Sammy Joelle Shirley Wrede, Dominique Rodil dos Anjos, Jan Patrick Kettschau, Horst Christoph Broding, Kevin Claassen

**Affiliations:** Faculty of Health Department of Human Medicine, Chair of Occupational Medicine and Corporate Health Management, Witten/Herdecke University, Witten, Germany

**Keywords:** Digital stress, Digitization, Public administration, Risk factors, Cluster analysis

## Abstract

**Objective:**

As the digitization of the working world progresses, the demands on employees change. Not least, this is true for the setting of public administrations in Germany, which is currently affected by the transformation to E-Government. This study aims to identify and describe a risk cluster of digitally stressed employees in public administrations.

**Methods:**

An online sample of 710 employees from three public administrations in North Rhine-Westphalia were surveyed about digital stress (7 items) and several potential risk factors (19 items) derived from the current research. In the first step, a hierarchical agglomerative cluster analysis is used to detect the risk cluster. This is followed by a comparison to the group of the remaining employees regarding their risk profiles.

**Results:**

The analysis states that the digitally stressed cluster accounts for approximately ten percent of the public administration’s employees of the total sample. Employees in the risk cluster are less satisfied with on-site work overall, experience less collegial support on-site, experience less collegial support in the home office, resign more often, are more likely to feel overwhelmed, are less educated, are older in age and more often have relatives in need of care.

**Conclusion:**

This work was able to identify and describe a group of digitally stressed rather than left-behind employees in public administrations to bring awareness to potentially destructive factors in the digital transformation process but eventually to social inequalities. The findings offer the basis for interventions to arise and evoke potential for further research.

## Background

The digitization of the working world has profound effects on the mental health of employees [1]. Digital stress occurring for individuals dealing with digital technologies is described as the "inability of an individual to deal with new technology in a healthy way, leading to stressful experiences" [2]. Studies are urgently needed that report on the clusters most affected by digitalization to benefit from technological advancements in the long term, to minimize its negative effects (which could result in detrimental organizational and health phenomena) and to support equal opportunities among workers. This work contributes to filling the gap by examining digital stress in German public administration.

Digitization is described by the German Federal Agency for Civic Education as a process that converts and stores information in machine-readable data and includes operations of data processing, transmission, and combination [3]. Piasecki (2020) specifies that digitization in the setting of public administration "essentially means shifting administrative tasks to a new digital level and integrating traditional (paper-based) processes into computer-based processing structures to optimize results and accelerate procedures" [4]. The usage of advanced technology profoundly affects the work environment and organization, which results in an "acceleration, increasing abstractness, flexibilization and individualization of processes and results" [5]. Work-related digitization is part of the transformation to the working world 4.0, in which routine steps are replaced by knowledge work with complex, dynamically evolving activities, thus changing the nature of office work. The expansion of existing technologies encourages mobile working at flexible workplaces and working hours [6]. Work 4.0 has become established in the German-speaking world as a term for the fundamental structural transformation in gainful employment resulting from advancing digitization [7]. Digital and mobile communication systems enable companies to collaborate and coordinate over greater physical distance and with flexible timeframes. It also facilitates increased access to expertise, specialist knowledge, and resources. The change in workplace opportunities results in a variety of new work models. Boundaries in different areas, e.g., between locations, companies, customers and workforces, are becoming increasingly blurred [8]. Routine activities are becoming increasingly automated, so employees’ tasks are becoming more cross-functional and cross-divisional, while work is increasingly information-based. Consequently, the targeted further qualification of the workforce in digital literacy is of crucial importance.

With the emergence of the SARS-CoV-2 pandemic in 2020 and the subsequent measures taken with the aim of containing the virus and preventing COVID-19, especially home offices for physical rather than social distancing as well as infection protection, digitization at the workplace has experienced a widespread push [9].

Simultaneously, digital training in the public sector lags behind other industries [10]. For the German federal state of North Rhine-Westphalia, the E-Government Act (EGovG NRW), including the e-file, was passed with the aim of modernizing public administrations and keeping them competitive and capable of taking action [11]. The model regions, which form the basis of this study, play a pioneering role. With i.a. digital services (business, housing subsidies, student grants, etc.), electronic proof of identity, cross-border standardization, Europe-wide usability, digitization of (high) schools and the digital citizens' office, financial savings are planned to be reached by 2025 [12]. Despite its potential, Germany ranks only 22nd in Europe in terms of the digitization of administration and the introduction of e-government services, according to the EU's Digital Economy and Society Index 2020 [13].

Digital competence is becoming increasingly important in a fast-paced environment, not least for maintaining the ability to work. Otherwise, there is a risk of individual overload that occurs when the demands exceed the available resources, e.g., the required competence for the work tasks [14, 15]. Karasek's job demand-control model (JDC) explains work-related psychological strain with a discrepancy between demands and the range of one's own opportunities for action (autonomy scope). An increase in work demands lead to a congestion of action energy, the release of which is dependent on one's own autonomy of opportunities for action and is either converted productively or leads to stated psychological strains [16]. The JDC model has been extended to the job demand control support model (JDCS) to highlight social support as an important resource [17]. From the underlying generalization to the job demand-resources model (JD-R), it can be concluded that less strain accompanied by optimal work motivation and performance results when demands and coping resources are balanced.

The following work builds upon the JD-R model, as all variables tested are classified as demands, resources or strains leading to the formation of a digitally stressed risk cluster.

Continuous overload at the workplace leads to negative health consequences on many levels. Theorell et al. (2015) work out a systematic connection between work environment conditions and symptoms of depression [18]. In another paper, Theorell et al. (2016) promoted a link between workplace conditions and the development of cardiovascular diseases [19]. Nixon et al. (2011) outline the psychosomatic effects of workplace stressors [20]. The included stressors were organizational constraints, interpersonal conflict, role conflict, role ambiguity, workload, work hours, and lack of control. All of the occupational stressors were significantly related to physical symptoms. Gastrointestinal complaints and sleep disturbances were significantly related to more stressors than the other symptoms examined. Work overload and role ambiguity are found to be the two most dominant stressors, whereas intrusive technology characteristics are found to be dominant predictors of stressors [21]. Galluch et al. (2015) confirm the effects of digital stress and are experimenting with matched interventions for digital stress management [22]. Smith et al. (1999) already addressed the resulting occupational stress in human-computer interaction due to increasing technology use at work [23]. Diebig et al. (2020) describe the extent of the psychosocial impact of digitization on health and work, on the macro to the micro level [24]. Körner et al. (2019) broke down the origin of perceived stress in human-machine interactions during work to technical conditions (malfunctioning and poor usability), how attentive or otherwise engaged users are (low situational awareness) and user competencies (increasing demands on employee skills) [25]. To maximize positive effects for individuals and the organization while minimizing negative consequences of digitization, Diebig et al. (2018) set content- and process-related requirements for the German risk assessment of mental stress in the context of Industry 4.0. The qualitative study includes updated definitions and data collection methods [26]. Turel et al. (2019) summarize the state of the art research on the "dark side of digitization of the individual" as follows: information system security behaviors, problematic and addictive use of technologies and loss of control over technology-mediated decisions, and technostress, loss of privacy and the blurring of work-life boundaries [27].

Gimpel et al. (2020) stated that in Germany, the increased work in the home office lengthens the periods in which work is done due to the intensified mixing of work and private life, resulting in the working time scheme as an interacting stressor [28]. This leads to the first of 19 hypotheses that are found (sometimes mingled together) after each paragraph:

*Employees within the risk cluster work full-time more often than part-time or on a marginal basis.*

In public administrations, the occupational group of civil servants takes up more than 30 percent [29]. Civil servants are not only stereotypically associated with "work by rule", coming along with recognition conflicts rather than internal resignation [30]. This becomes relevant regarding the employees’ ability to act and adapt. Workers with management responsibility are more accustomed to digital work because they have acclimated to digital work, as these workers might be field service employees and employees who already worked in a home office before the pandemic. People with experience or confidence in dealing with digital technologies and media cope better with the digital workplace situation [28].

*Employees within the risk cluster are more often civil servants, are less likely to have managerial responsibilities, were (prior to the COVID-19 pandemic) less likely to be in a home office and are less likely to work in the field.*

A lower level of general satisfaction is assumed for this group with the goal of identifying and describing a risk cluster of employees in German public administration who suffer more often from self-reported digital-related difficulties, due to the overall disadvantaged situation [31].

*Employees within the risk cluster are less satisfied with the overall on-site and home office work.*

It is assumed that in the home office, employees find less support not only by colleagues and supervisors but also within their own households, as many family members are equally affected by digital stress and expanding working hours. The effects are evident in an increased number of work-home conflicts resulting in feelings of overload and ultimately resignation. Social support, however, is associated with higher work ability and less disease [32]. More specifically, social support acts as a mediator between work stress and depression [33]. To analyze whether support resources are in line with new working conditions, technical and social support must be considered on site as well as in the home office.

*Employees within the risk cluster feel less technically supported on-site, feel less technically supported in the home office, experience less collegial support on-site, experience less collegial support in the home office, resign more often and are more likely to feel overwhelmed.*

There are several challenges arising when designing workplace interventions addressing the work ability of older employees [34]. However, when Gimpel et al. (2018) examined "digital stress in Germany", they concluded that digital stress is more pronounced among 25- to 34-year-old workers than among other age groups [2]. With regard to gender, they emphasize that women worked in further digitalized workplaces and at the same time experienced a higher level of digital stress than men. Ragu-Nathan et al. (2008) [35] and Trafadar et al. (2011) [36] indicate that men experience more digital stress than women based on survey data from the United States. Current European studies fit the contrary results, similar to Gimpel et al. (2018) [2], who found that "zoom fatigue" is significantly more prevalent among female workers [37]. Following Ragu-Nathan et al. (2008) [35], perceived digital stress decreases not only with increasing age but also with level of education and job experience.

*Employees within the risk cluster are more likely to be female, are less educated and younger in age.*

Parents bear a somewhat higher burden [28]. While children represent a context-dependent stressor in terms of employees' individual resource capacity, they could also promote intergenerational exchanges with "digital natives" [38]. Caring for relatives, on the other hand, represents a risk factor, as it is probably associated with fewer resources available for gaining digital competence.

*Employees within the risk cluster are more likely to have children in the household and more often have relatives in need of care.*

## Method

All in all, it is assumed that the employees within the risk cluster... (see Table 1).

**Table 1:** Hypotheses to be tested

Hypothesis	Label
1. work full-time more often than part-time or on a marginal basis.	Working time
2. are more often civil servants.	Civil service
3. are less likely to have managerial responsibilities.	Management responsibility
4. were (prior to Covid-19) less likely to be in a home office.	Home office
5. are less likely to work in the field.	Field service
6. are less satisfied with the on-site work overall.	Satisfaction on site
7. are less satisfied with the home office work overall.	Home office satisfaction
8. are contacted more often outside of work hours.	Contact free time
9. feel less technically supported on-site.	Technical support on site
10. feel less technically supported in the home office.	Technical support in home office
11. experience less collegial support on-site.	Social support on site
12. experience less collegial support in the home office.	Social support in the home office
13. resign more often.	Resignation
14. are more likely to feel overwhelmed.	Excessive demands
15. are more likely to be female.	Gender
16. are less educated.	Education
17. are younger in age.	Age
18. are more likely to have children in the household.	Children
19. more often have relatives in need of care.	Care

The hypotheses are tested to evaluate which factors are associated with digital stress in public administrations. While 17 of the 19 hypotheses refer to risk or (context-dependent) resource factors, feeling overwhelmed as well as resignation (13.+14.) have been added as consequences of digital stress and are regarded as psychological strain. The last five hypotheses refer to sociodemographic attributes.

The data basis for the hypotheses tests is a cross-sectional study that was funded as part of the project "Health and Digital Change" (GudW). The study itself was given a positive vote by the Ethics Committee of the University of Witten/Herdecke under reference number 158/2020, i.e. it was checked for compliance with the Declaration of Helsinki on Medical Research Involving Human Subjects and with the applicable data protection regulations.

The primary data were collected online from n = 710 employees in three German municipal administrations of the project-related “digital model regions” in North Rhine-Westphalia. These represent the federal state with a rural region, a metropolitan area and an international border region. All employees of departments with ongoing digital implementation processes (mainly introduction of the e-file) received an invitation link via the internal mail system of the administrations. Thereby, their volunteering circa 15-minute participation and their consent to scientific processing and publication were asked. After 14 days of no response, a single reminder was sent. The invitation link led to an external survey platform based on the software “LimeSurvey” hosted by Witten/Herdecke University to address privacy concerns.

Considering the sampling size of 1,319 invited employees and the subsequent response rate of RR = 0.54, an online bias is not assumed. The participants primarily worked in the departments of housing and social services (29%), human resources and organization (17%), security and construction (15% each). 59.12% of them were female, 73.89% worked in full-time, 38.46% were civil servants, 19.69% had management responsibilities, 48.57% had obtained a university degree, 40.33% currently had children in their household and 38.62% had to take care of relatives. Mean age was 44.57 ± 12.69 years. The first results regarding further outcomes as well as further information on the sample can be found in Claassen et al. (2021) [39]. Thus, this is already a multioutcome study in its approach.

Missing values due to item nonresponse were imputed for the present analysis using multivariate imputation by chained equations (MICE) following Rubin [40]. The first imputed dataset without a stochastic component was used. This was followed by a hierarchical agglomerative cluster analysis using complete linkage to identify the risk cluster [41]. The distance matrix was based on Euclidean distances, called the L2 norm.

To validate the quality of the cluster solution, a scree plot was used. The optimal number of clusters is the difference between the number of cases to be clustered and the fusion step, after which the distance between two observations ("height" on the Y-axis) increases abruptly.

The second step is a descriptive comparison of the risk cluster to the group of the remaining employees. Here, the ordinal items of the standardized Likert scale are treated quasimetrically. Therefore, the arithmetic mean is reported. For dichotomous and nominal variables, the proportion value is reported. Significance testing for differences is performed using the two-sample t tests for means and Fisher's exact test for proportions, for each of which the p value is reported. The associated significance level as the basis for the test decision for the formulated hypotheses is α = 0.05. Although we conducted statistical testing as a rough indicator of relevant variables, the study is explorative.

The variables were selected based on constructs of mental stress and new ways of working by the Joint German Occupational Safety and Health Strategy [42]. The item formulations were finalized in a group discussion with two project leaders of each of the participating administrations, who were either medical officers (specialist in occupational medicine) or occupational health managers (master’s degree).

The seven digitization-related variables representing the basis of the cluster analysis differ from the variables used to describe risk factors. The former variables predominantly ask about agreement on a scale of 1 (“strongly disagree”) to 4 (“strongly agree”) or about stressfulness. The latter variables relate to work-specific and sociodemographic items, as well as to the frequency of negative emotional states (feeling overwhelmed, resignation). Analytically, the digitization-related items were not summarized to a score, as they do not rely on a standardized validated instrument but act as separate clustering variables. However, we assessed their internal consistency via Cronbach’s α. A presentation of the two groups of variables with the corresponding question formulation and response coding is shown in Tables 2 and 3.

**Table 2:** Digitization-related cluster variables

Item	Coding	Label
Dealing with digital applications at work is easy for me.	1 = does not apply 2 = rather does not apply 3 = rather applies 4 = very much applies	Digi1
The increasing digitization in the public administration has no negative impact on my health.	1 = does not apply 2 = rather does not apply 3 = rather applies 4 = very much applies	Digi2
I feel well prepared for digitization by my employer.	1 = does not apply 2 = rather does not apply 3 = rather applies 4 = very much applies	Digi3
I support the switch to digital applications at my work.	1 = does not apply 2 = rather does not apply 3 = rather applies 4 = very much applies	Digi4
Digitization leads to...	1 = more work 2 = just as much work 3 = less work	Digi5
How stressful do you find constant screen work?	1 = stressful 2 = rather stressful 3 = rather not stressful 4 = not stressful	Digi6
How stressful do you find the need to be available via different communication channels at the same time?	1 = stressful 2 = rather stressful 3 = rather not stressful 4 = not stressful	Digi7

**Table 3:** Work-specific, sociodemographic, and emotional variables

Item	Coding	Label
Do you work full-time, part-time or are you marginally employed?	1 = full time 2 = part time 3 = marginally employed	Working time
Are you a civil servant or an employee covered by collective bargaining agreements?	1 = civil servant 2 = employed according to collective agreement	Civil service
Do you have management responsibility?	1 = yes 2 = no	Management responsibility
How often did you work in a home office before Corona?	1 = never 2 = occasionally 3 = predominantly 4 = always	Home office
How often do you work in the field?	1 = never 2 = occasionally 3 = predominantly 4 = always	Field service
How satisfied are you with on-site work overall?	1 = not satisfied 2 = rather not satisfied 3 = rather satisfied 4 = satisfied	Satisfaction on site
How satisfied are you with home office work overall?	1 = not satisfied 2 = rather not satisfied 3 = rather satisfied 4 = satisfied	Home office satisfaction
How satisfied are you with work-related contact outside of official work hours?	1 = not satisfied 2 = rather not satisfied 3 = rather satisfied 4 = satisfied	Contact free time
How satisfied are you with on-site support in case of technical difficulties (hardware/software issues)?	1 = not satisfied 2 = rather not satisfied 3 = rather satisfied 4 = satisfied	Technical support on site
How satisfied are you with the support in case of technical difficulties (hardware/software problems) in the home office?	1 = not satisfied 2 = rather not satisfied 3 = rather satisfied 4 = satisfied	Technical support in home office
How satisfied are you with the possibility to get on-site support from colleagues if needed?	1 = not satisfied 2 = rather not satisfied 3 = rather satisfied 4 = satisfied	Social support on site
How satisfied are you with the possibility of receiving support from colleagues in the home office when needed?	1 = not satisfied 2 = rather not satisfied 3 = rather satisfied 4 = satisfied	Social support in the home office
How often do you feel resigned?	1 = always 2 = frequently 3 = rarely 4 = almost never	Resignation
How often do you feel overwhelmed?	1 = always 2 = frequently 3 = rarely 4 = almost never	Excessive demands
With which gender do you identify yourself?	1 = female 2 = male 3 = diverse	Gender
What is your highest level of education?	1 = no degree 2 = secondary school diploma 3 = secondary school leaving certificate 4 = advanced technical college entrance qualification 5 = general university entrance qualification 6 = university degree	Education
How old are you in years?	years (numeric)	Age
Are there children living in your household?	1 = yes 2 = no	Children
Are there other relatives you have to take care of?	1 = yes 2 = no	Care

## Results

**Figure 1:**
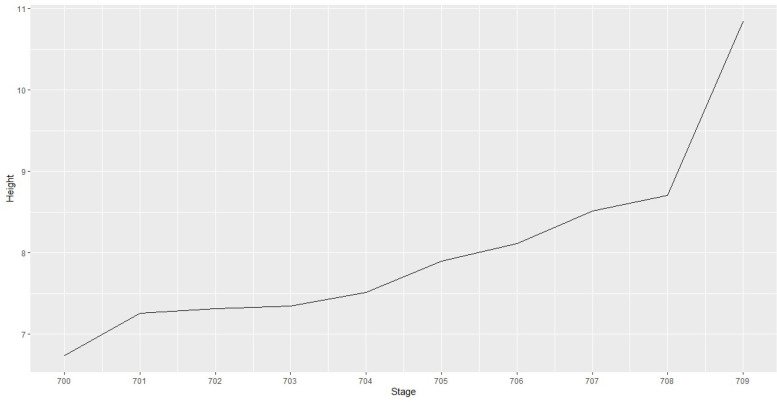
Scree plot for the risk cluster

The results of the cluster analysis confirm a two-cluster solution so that the assumed assignment to a (single) risk cluster can be confirmed empirically. In fact, the scree plot in Figure 1 shows that the distance increases sharply after fusion step 708.

Sixty-five of 710 respondents, or 9.15 percent of respondents, were found within the identified risk cluster. A description of this cluster in comparison to the nonrisk group (including p values) for the digitization-related cluster variables can be found in Table 4 and for the descriptive variables in Table 5. Significant variables are marked bold. In this context, the digitization-related cluster variables show an acceptable internal consistency of α = 0.73. The items are sufficiently interrelated, and approximately three-quarters of the construct’s total variance is not due to chance.

**Table 4:** Group comparison of digitization-related cluster variables

**Label**	**Risk-cluster**	**Remaining employees**	**p value**
**Digi1 (mean)**	**2.75**	**3.42**	**< 0.001**
**Digi2 (mean)**	**1.92**	**3.28**	**< 0.001**
**Digi3 (mean)**	**1.80**	**2.43**	**< 0.001**
**Digi4 (mean)**	**2.20**	**3.37**	**< 0.001**
**Digi5 (percentage** **"more work")**	**0.66**	**0.22**	**< 0.001**
**Digi6 (mean)**	**1.83**	**2.74**	**< 0.001**
**Digi7 (mean)**	**1.97**	**3.18**	**< 0.001**

**Table 5:** Group comparison of work-specific, sociodemographic, and emotional variables

**Label**	**Risk-cluster**	**Remaining employees**	**p value**
Working hours (percentage "full-time")	0.69	0.74	0.41
Civil service (percentage "yes")	0.47	0.38	0.16
Management responsibility (percentage "yes")	0.27	0.19	0.17
Home office (percentage at least "occasionally")	0.18	0.15	0.23
Field service (percentage at least "occasionally")	0.43	0.47	0.35
**Satisfaction on site (mean)**	**2.80**	**3.28**	**0.02**
Satisfaction in home office (mean)	3.39	3.16	0.05
Contact free time (mean)	2.90	3.28	0.08
Technical support on site (mean)	2.48	2.87	0.08
Technical support in home office (mean)	2.74	2.94	0.29
**Social support on site (mean)**	**2.96**	**3.33**	**0.02**
**Social support in home office (mean)**	**2.77**	**3.21**	**< 0.01**
**Resignation (mean)**	**1.85**	**2.21**	**< 0.01**
**Excessive demands (mean)**	**1.77**	**2.40**	**< 0.001**
Gender (percentage "female")	0.61	0.59	0.71
**Education (share "university degree")**	**0.35**	**0.50**	**0.03**
**Age (mean)**	**48.76**	**44.13**	**0.02**
Children (percentage "yes")	0.35	0.41	0.37
**Care (share "yes")**	**0.53**	**0.37**	**0.02**

Significant differences at the five-percent level between the two groups of employees emerge for all digitization-related variables, which form the basis of the successful cluster analysis. Employees within the risk cluster are viewed as digitally stressed. This means, on average, that they support the switch to digital applications significantly less often because they do not handle the transition well and simultaneously feel ill prepared by their employer. For them, work-related digitization subjectively leads to additional work. They feel stressed by constant screen work as well as by digital availability demands and expect adverse health effects. Based on this clustering, significant risk factors can be extracted.

As opposed to their digitally less stressed colleagues, administrative employees within the risk cluster show significantly less work satisfaction and lack social support by colleagues (on-site as well as in the home office). They resign more often, are more likely to feel overwhelmed, are less educated and more often have relatives in need of care. On average, they are also older in age so that the assumed direction is reversed.

On the other hand, there are no significant differences regarding working hours scheme, status as a civil servant, management responsibilities, home-office frequency, field service, work-related contacting outside of official working hours, technical support (on-site as well as in the home office), gender and children in the household. Overall satisfaction in the home office fails to reach significance at p = 0.05, but it is noticeably higher within the risk cluster than among the remaining employees.

Accordingly, Hypotheses 6, 11, 12, 13, 14, 16, 17 and 19 receive confirmation, while the remaining hypotheses have to be rejected.

## Discussion

This study is one of the first of its kind to evaluate digital stress factors in the context of public administrations. As a result, the cluster of digitally stressed employees accounts for approximately 10 percent of the public administration’s employees in relation to the total sample. Resigning and feeling overwhelmed are regarded as significant stress consequences or strain variables themselves. This indicates that it is of crucial importance to intervene with targeted support to relieve the strain to prevent more far-reaching adverse health effects, as stated in the introduction. On the other hand, a lack of job satisfaction on-site, social support and (high) school education, as well as older age and relatives in need of care, can be identified as risk factors for digitization-related stress.

In particular, the key role of social support fits the corpus of the existing literature. Regarding emotional exhaustion, anxiety and depression, social support acts as a mediating protective factor [43]. Lecca et al. (2020) focus on social support interventions as a tool to manage work-related stress itself [44]. Ambiguities remain due to measurement heterogeneity and evidence of reverse causality biases, as Gariépy et al. (2016) point out [45].

All other describing assumptions, such as those concerning technical support, job position and engagement, gender or having children, cannot be confirmed. They do not show any significance as variables of this cluster. However, higher satisfaction in the home office of the risk cluster is almost significant, interestingly in the opposite direction to the hypothesis. This suggests that the digitally stressed cluster is content to have the opportunity to work from home to better balance work and family (e.g., to care for relatives, which applies to more than half of the employees within the risk cluster). This could indicate that the risk cluster is in fact digitally stressed rather than left behind.

Contrary to the prior assumption, employees in the risk cluster are older, which might be traced back to a possible inferiority regarding digital competencies acquired off the job. Apart from that, it could be a limitation that the length of the time employees have already spent on the job is not recorded. In addition, no distinction is made between working at home and from different mobile locations. Familiarity with mobile working is likely to be associated with more digital affinity.

Furthermore, it must be kept in mind that the respondents subjectively assessed themselves on a four-point Likert scale. Although less frequently than scales with an uneven number of normally five points, even pointed Likert scales are commonly used to “force” respondents to a nonneutral decision [46]. This was attenuated by the fact that the respondents always had the option to not answer and skip questions.

Due to the cross-sectional study design and because potential third variable effects as well as their interactions were not controlled for, causation could not be established. Furthermore, the sample is not fully representative of public administrations as a whole for two reasons that could potentially lead to selection bias: i) the voluntariness of participation and ii) the necessity to include only employees from departments with ongoing digital implementation processes. Consequently, it is once again to be highlighted that this study is explorative in its nature.

As the study at hand addresses a rather new research topic and the survey questions were formulated in cooperation with the practitioners of the public administrations, no standardized instruments were used. Hence, the reliability and validity of the results could be limited. Some theoretic constructs are covered only in approximation. For example, job satisfaction is a multifaceted construct [47]. A unidimensional overall measurement (of on-site versus home-office satisfaction) lacks the precision that would be needed in a confirmative study. Targeting social support, the focus is on the collegial dimension, factoring out social support by supervisors.

The study only looked at employees in public administrations. Therefore, transferability to computer workstations in general remains questionable. As opposed to the free economy as well as to public administrations in other countries, German public administrations and their employees might be viewed as overly bureaucratic and change resistant [48]. It will therefore be interesting to see how high the proportion of digitally stressed employees is estimated to be in other countries and different economic sectors based on representative surveys. Further research should aim to confirm which factors are stressors, moderators, mediators, and resources and which are consequences of psychological stress in a more complex multivariate causal model.

However, with this study, we managed to identify and describe a group of digitally stressed employees in public administrations, not least to bring awareness to potentially unhealthy factors of the digital transformation process – and eventually to social inequalities as well. Based on our findings, we promote the following initial approaches for the practical primary prevention of stress-related illnesses and absences from work. Additionally, they might also improve the attractiveness of employers. These approaches offer the additional potential to prevent a digital divide early in the process and to maintain the working ability of digitally stressed employees in the long term, which is societally and economically desirable.

Due to the high level of satisfaction within the home office setting, the expansion of home office options represents an impetus for action, while digital leadership as well as leading on distance skills by executive managers have to be developed further. Simultaneously, a transformation from mobile working to fixed home-based telecommuting workstations with the associated German legal implications for ergonomic equipment as well as workplace health promotion could nudge positive effects. For a distinction of telecommuting options, see Pearce (2009) [49]. Another sensible intervention derived from the results of this study could be the appointment of "digital pilots" for social support [50]. Future concepts might transfer the idea of digital pilots to a working world that is, independent of COVID-19 increasingly shaped by phases of physical distance. Moreover, the expansion of social care advisory services, either as employee assistance programs or as a structural offer in the company setting, could be supportive, especially for employees with relatives who need care – taking care of those who are taking care. This could simultaneously increase the employer’s attractiveness and branding, which is not least vital in public administrations.

## Data Availability

The dataset supporting the conclusions of this article is included within the article. Data available on reasonable request through the corresponding author SW.
